# Opportunities and challenges of using metagenomic data to bring uncultured microbes into cultivation

**DOI:** 10.1186/s40168-022-01272-5

**Published:** 2022-05-12

**Authors:** Sijia Liu, Christina D. Moon, Nan Zheng, Sharon Huws, Shengguo Zhao, Jiaqi Wang

**Affiliations:** 1 State Key Laboratory of Animal Nutrition, Institute of Animal Sciences, Chinese Academy of Agricultural Sciences, No. 2 Yuanmingyuan West Road, Haidian, Beijing, 100193 China; 2 College of Pastoral Agriculture Science and Technology, Lanzhou University, Lanzhou, 730020 China; 3 AgResearch Ltd., Grasslands Research Centre, Palmerston North, New Zealand; 4 School of Biological Sciences and Institute for Global Food Security, 19 Chlorine Gardens, Queen’s University Belfast, Belfast, UK

## Abstract

Although there is now an extensive understanding of the diversity of microbial life on earth through culture-independent metagenomic DNA sequence analyses, the isolation and cultivation of microbes remains critical to directly study them and confirm their metabolic and physiological functions, and their ecological roles. The majority of environmental microbes are as yet uncultured however; therefore, bringing these rare or poorly characterized groups into culture is a priority to further understand microbiome functions. Moreover, cultivated isolates may find utility in a range of applications, such as new probiotics, biocontrol agents, and agents for industrial processes. The growing abundance of metagenomic and meta-transcriptomic sequence information from a wide range of environments provides more opportunities to guide the isolation and cultivation of microbes of interest. In this paper, we discuss a range of successful methodologies and applications that have underpinned recent metagenome-guided isolation and cultivation of microbe efforts. These approaches include determining specific culture conditions to enrich for taxa of interest, to more complex strategies that specifically target the capture of microbial species through antibody engineering and genome editing strategies. With the greater degree of genomic information now available from uncultivated members, such as via metagenome-assembled genomes, the theoretical understanding of their cultivation requirements will enable greater possibilities to capture these and ultimately gain a more comprehensive understanding of the microbiomes.

Video Abstract

**Supplementary Information:**

The online version contains supplementary material available at 10.1186/s40168-022-01272-5.

## Introduction

Microbes are the earliest known life forms on earth, and fossil evidence for their existence dates to over 3 billion years ago. Subsequently, microbial life has diversified and adapted, and is found in almost all environments examined [1–3]. These include soils and oceans, and also host-associated environments such animal gut systems or the rhizospheres of plants, which they have co-evolved with and become dependent upon for host health and function. Microbes possess enormous metabolic and physiological versatility and are essential to virtually all biogeochemical cycling processes [4]. Furthermore, microbiota-host associations are widespread in nature [5, 6], and the host can benefit from the association in different ways. Microbes may aid in assimilating or synthesizing essential nutrients catabolize otherwise indigestible substrates, or act as a source of nutrition for the host themselves. They can also protect their hosts from pathogens and toxic substances or regulate host physiology, including immunity, development, and even the host’s social behavior [7–12]. Advances in DNA sequencing technologies have transformed our understanding of the extent of the diversity of microbial life on earth [13–15], particularly that of the prokaryotic bacteria and archaea. Such studies have, however, also highlighted the extent to which the majority of species, including major microbial lineages, have not yet been brought into cultivation [16, 17]. Consequently, most of our understanding of the microbial world is either derived from the minority of cultured species or from data generated from culture-independent studies. Although sequencing technologies have provided significant new insights into microbial diversity and function, obtaining cultured representatives of key uncultured lineages is critical to directly assess their metabolic and physiological functions, and hence gain a greater understanding of their biology and ecological roles in their natural environments.

Efforts to bringing uncultivated microbial “dark matter” into cultivation have been growing in popularity in the past decade as the value of cultures in the post-‘omics’ era is increasingly recognized. Traditional untargeted and new high-throughput methods of cultivation, such as culturomics platforms that rely on the use of ranges of culture media and high-throughput screening approaches, have resulted in the cultivation of many new and previously uncultured lineages being brought into culture [18, 19]. Such methods, are often labor and resource intensive, and may not necessarily result in the capture of specific target microbial groups of importance within the community. Genetic data from uncultured organisms of interest therefore holds significant promise to aid their cultivation. Indeed, culture-independent data, such as those from metagenome sequencing and single-cell genomics studies, have broadened the potential for targeted microbial isolation through identifying tailored strategies based on unique attributes of the target organisms for efficient isolation. Such methods hold great promise for tapping into the rich biological and genetic resources that uncultured microbes represent [20]. In this paper, we provide an overview of progress in methods for metagenome-guided microbial isolation to further characterize new microbial species.

### The uncultured majority

Much controversy has been associated with attempts to estimate the global diversity of microbes on earth [15, 21–25]. While various estimates have been made [15], recent estimates of bacterial and archaeal diversity based on global 16S rRNA gene sequence datasets have predicted the existence of 2.2–4.3 million prokaryotic operational taxonomic units (OTUs; clustered at 97% similarity), that are akin to species-level taxa. It is likely that the global number of prokaryotic ecotypes greatly exceeds these estimates due to genomic and phenotypic diversity data that is not captured in such surveys [26]. Moreover, important microbial groups such as fungi, protozoa, and other single-cell eukaryotes are often overlooked in diversity estimates, but contribute significantly to the function of microbial ecosystems. In contrast to the diversity of microbial life, many of the bacterial species in cultivation are from four bacterial phyla (*Bacteroidetes*, *Proteobacteria*, *Firmicutes*, and *Actinobacteria*) [27–29]. These phyla are dominant in gut microbial communities, which may reflect the intense interest in the study of the human gut microbiota as compared those of different environments [17]. Moreover, it has been estimated that uncultured genera and phyla could comprise 7.3 × 10^29^ (81%) and 2.2 × 10^29^ (25%) of microbial cells across earth’s microbiomes, respectively [17]. Across different, non-human environments, uncultivated species are among the most dominant organisms present and are assumed to have key ecological roles, thus insight into their biology is important to understand their contributions to these ecosystems. In the last 10 years, progress in the isolation and cultivation of microorganisms from a range of environments has been is slow because there are many complex factors that are not well understood that pose barriers to cultivation in the laboratory. Natural environments for microbial growth are often complex, and vary in parameters such as pH, temperature, and pressure. It is also difficult to mimic strict nutritional requirements, and growth factors required that are unknown. Moreover, some microbes grow under anaerobic and other extreme conditions, which require specialist facilities to replicate under laboratory conditions [30, 31]. Microorganisms may also exist in a dormant state may in nature [32], which needs to be overcome to enable growth in the laboratory . In some cases, microbes require cross-feeding or close interactions with the other host or other community members [33, 34]. There are also some bacteria with very low abundance in the environment or some rare species [35], which require selection or enrichment strategies to capture, or robust screening assays to identify. This is more challenging for slow growing bacteria, which may not be able to compete with fast-growing species when isolated in vitro [34], so their enrichment is further a challenging task.

### The importance of cultivation in the age of omics

Stable pure cultures of microbes are valuable resources that can be used to experimentally investigate microbial traits [36], confirm the activities of novel genes through functional characterization studies, thereby improving the accuracy of gene annotations [37]. Cultures can also be used to generate reference genomic data [38, 39] that can be further used to interpret microbial community function through metagenome and metatranscriptome analyses. Moreover, the availability of cultures enables new possibilities for applications for a variety of outcomes including health and industrial.

Cultures enable microbial metabolism to be studied at the biochemical level, and reveal as-yet unknown physiological traits under different growth conditions [40]. These features are difficult to infer from genomic data as it is not known which genes are expressed, the interactions between their gene products, and how this is affected under different environmental conditions. Novel biochemical pathways and enzymatic reactions have been found through experimental analyses of cultured microbes [41, 42], which were not apparent by genome analyses alone [42], were found by culturing and analysis. Although meta-omics can undoubtedly provide considerable insights into the biology of the uncultured majority and enable new hypotheses to be generated, live cultures are invaluable resources to experimentally test such hypotheses and enable experimental validation of phenotype and ecology, thus widespread efforts are being made to bring more microbial species into cultivation [43]. Furthermore, multi-species interactions, evolutionary principles, population dynamics and pathogenicity can only be experimentally validated when isolates are available [40]. In addition, the availability of cultures allows their potential in applications such as industrial process, probiotics, seed inoculants, to be harnessed. Culture collections represent biobanks repositories that enable resources to be shared, and used for biotechnology discovery.

The availability of cultures will contribute to the richness of reference databases and taxonomic frameworks that will further aid biological interpretation of microbial function in ecosystems. Currently in genetic databases, such as KEGG [44], almost half of the genes present are of undetermined function, and it is recognized that many annotations are not accurate [45]. The functional validation of microbial genes *via* experimental data will considerably aid the extent and accuracy of annotations, which could further enhance the ability to bring more uncultured microbes into cultivation. Moreover, classifying organisms is central to improving taxonomic frameworks to describe biodiversity. Presently, the International Code of Nomenclature of Prokaryotes recognizes only cultures as ‘type material’, thus uncultured organisms cannot be formally named. While community efforts to change this position are underway to recognize DNA sequences as type material [46], new species cannot be formally described taxonomically without a cultured representative. This brings about disparity between taxonomic frameworks and recognition of uncultured organisms, which alternatively, it has been proposed that a nomenclatural code for uncultivated prokaryotes be developed to integrate the taxonomy of uncultured organisms into existing frameworks [46].

It is not always possible of obtain stable pure cultures of microbes, and enrichment cultures and stable defined co-cultures are also valuable to gain biological insights into the uncultured organisms. Enrichment cultures [47] enhance the population density of a particular group of microorganisms within the total microbial population of a sample. This is achieved by preferentially stimulating the growth of the target microbes by manipulation of the growth conditions. Co-culturing of two or more strains may be necessary for growth of the target organisms if they are dependent on another microbe for growth, such as via cross-feeding of metabolic substrates of one organism as substrate for the other. Enrichments and co-cultures can be used to further develop optimal growth conditions to isolated strain, or for routine culture maintenance.

### Prediction and reconstruction the metabolic pathways from metagenomic data

Metagenomics is the study of the collective genomes of the members of a microbial community, and can provide valuable insights for environmental uncultured microbes [48]. Metagenome sequencing is generally undertaken using a shotgun sequencing approach which is non-discriminant and can enable assignment of taxonomy and organism quantification to the species level, as well as allowing functional assignment to genes that are identified. A critical step in analysis is metagenome sequence assembly, a stitching together of individual DNA sequences [49]. Metagenome-assembled genomes (MAGs) are generated by binning assembled contigs, with similar characteristics, and quality filtering [50, 51]. MAGs therefore may represent a microbial genome of highly similar microbial strains.

Short-read (e.g., 100-150 bp) next generation sequencing (NGS) technologies, such as Illumina sequencing, are popular due to their high-throughput [52] and cost-effectiveness. Microbial genomes can be assembled from short-read sequencing data, but the assembly contiguity of these metagenome-assembled genomes is constrained by repeat elements [53] which may be overcome by some degree by long insert paired-end approaches. The recovery of MAGs is furthermore hampered by the presence of closely related strains. In contrast, long sequence reads can span entire common repeat elements, such as 16S rRNA genes, miniature inverted repeat transposable elements, transposons, gene duplications, and prophage sequences, and can improve assemble MAG quality considerably. Advances in sequencing technologies where long-read methods, such as single-tube long fragment read (stLFR) [54], PacBio [55, 56], and Nanopore [57] have been applied. These technologies have made it possible to reconstruct complete or single circular genomes from soils, freshwater lake and human stool samples. While MAGs have become a popular and near standard analysis for metagenome datasets [58, 59], they have been criticized for issues resolving 16S rRNA genes and biosynthetic gene clusters (BGCs), potential contamination, chimerism, loss of synteny and missing genes, lack of ability to distinguish multiple chromosomes, and extrachromosomal elements. However, MAG quality is being enhanced by use of long-read sequencing technologies as well as new binning algorithms that can result in complete circularized MAGs [60], thus enhancing their value for functional interpretation and metabolic reconstruction.

While not based on metagenome sequencing, single-cell genomics, the recovery of genome information amplified from single cells from environmental samples [61, 62], is also worthy of mention in this review as it is another culture-independent approach that yields genetic insights into the uncultured majority. This technology has reached a mature stage where now, hundreds of high and medium quality microbial single-cell amplified genomes (SAGs) can be readily obtained in a high throughput fashion [61]. Analysis of these genomic data can also guide the cultivation of as yet uncultured organisms.

The functional annotation of MAGs and SAGs is critical for metabolic pathway reconstruction and understanding the functional potential of organisms that can be used to aid their cultivation. In general, metagenome functional annotation involves two steps: gene prediction and gene annotation [63]. Coding sequence function is inferred based on its similarity to genes present in reference databases, or via hidden Markov models (HMMs) of functional domains, e.g., Pfam [64]. A range of databases such as KEGG pathways [65], EggNOG [66], dbCAN [67], RGI [68], Gene Ontology (GO) terms [69], COG [70], MetaCyc [71], BioCyc [72], Brenda [73], Rhea [74], EcoCyc [75], can be used to revealed the functional categories, protein domains, metabolic potentials and traits [76] of uncultured organisms.

Based on reference pathways which are available in public repositories, protein sequences can be mapped by sequence homology, which can facilitate the prediction and reconstruction the metabolic pathways. Present methods to reconstruct metabolic pathways include BlastKOALA [77], KAAS [78], GhostKOALA [77], and RAST [79]. However, there are still many metabolic pathways that remain uncharacterized, or are incomplete where some components are not identified [80]. In addition, these methods cannot predict new reactions or enzymes that do not exist in the reference pathways. In order to overcome the shortcomings of the above methods, it is necessary to have strong evidence on genome context association, such as gene-gene interactions [81], classification, and clustering based on their function and phylogenetic profiling [82]. Interpretation of such data to identify nutritional or conditional requirements to aid the isolation and cultivation of uncultured organisms is a momentous task and remains a significant challenge.

Based on other culture-independent genomic information, a number of strategies can be pursued to bring uncultured organisms into cultivation in a targeted fashion, as described below. These strategies contrast and complement many untargeted and high throughput methods to bring organisms into cultivation, such as Culturomics [19, 83], in situ culture [84, 85], single-cell isolation [86, 87], iChip [88], which have garnered many cultivation successes from different environments. The advances in metagenomic data analysis methodologies and novel cultural strategies provides a new opportunity to identify microbes/functions of interest and determine targeted approaches to isolate these.

### Strategies of metagenomic date guided microbial isolation

#### Culture medium optimization

**Fig. 1:**
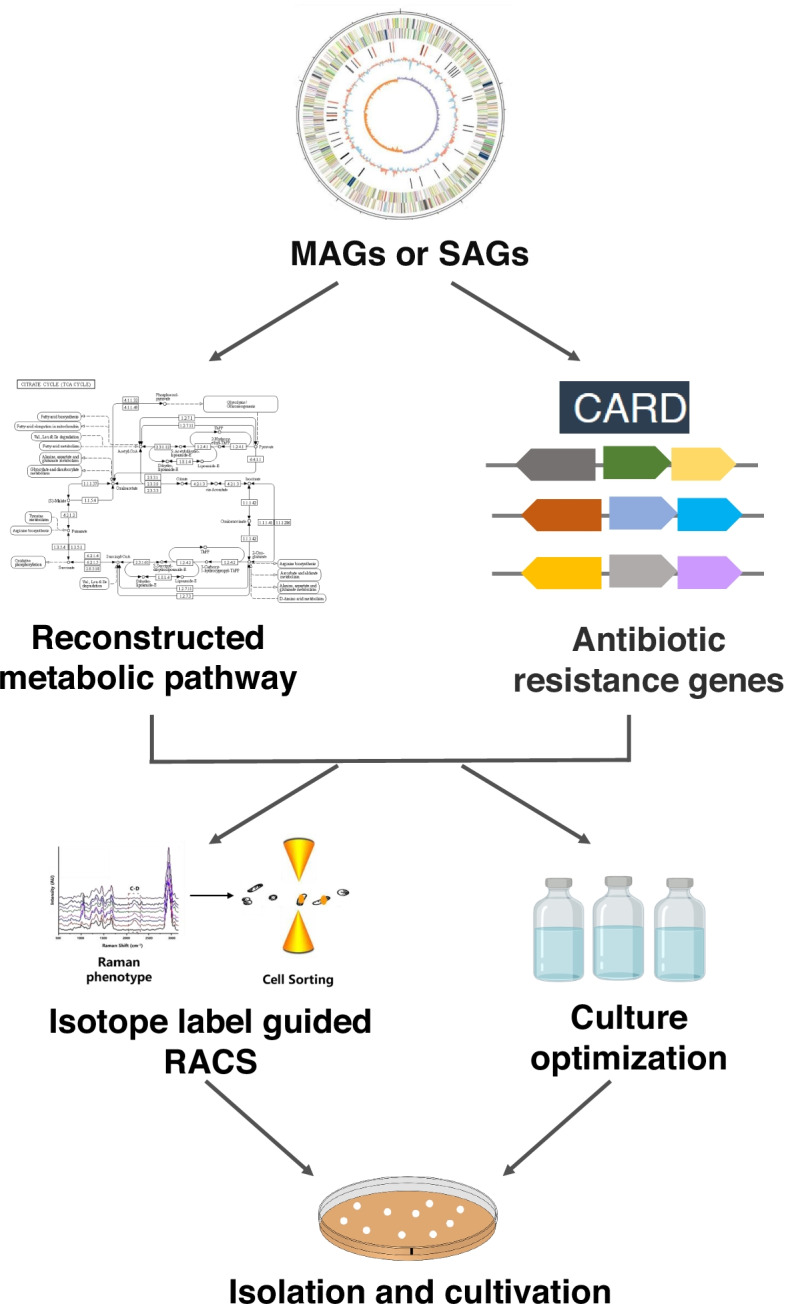
Illustration the methods of special medium design to isolatetarget microbiota

An understanding of nutritional requirements and metabolic characteristics is important for the isolation and stable cultivation of uncultured microbes. Metagenomic sequence data provides valuable information on traits such as primary metabolism, substrate utilization, oxygen requirements, resistance to antibiotics, and even potential interactions with the host through eukaryotic-like proteins [89], which provide opportunities to define culture media and growth conditions for fastidious microbes (Fig. 1). Translating genomic data to gain an understanding of the target organism’s physiology is not straightforward, however. This generally requires an in-depth knowledge of the relevant metabolic pathways and physiology for interpretation. Nonetheless, these methods have been successfully applied to isolate and culture novel microbes in different environments, including the ocean and gut.

In a pioneering study by Tyson et al, a *Leptospirillum* strain involved in nitrogen fixation was successfully isolated through improved culture media design informed by metagenomic data from an acid mine drainage biofilm [90]. The genus *Leptospirillum* had been divided into three groups—I, II, and III, based on 16S rRNA gene analyses. However, prior to this study, representatives of groups I and II were identified [91], but no cultured representatives of group III had been obtained. Tyson et al. [90] detected a single *nif* gene operon in the genomic data from the *Leptospirillum* group III population, which was lacking in group II. They inferred that *Leptospirillum* group II, also present in the biofilm, lacked nitrogen-fixing genes. So, the growth medium was modified to lack nitrogen and was used to successfully isolate the first cultivated representative of *Leptospirillum* group III based on the ability to carry out nitrogen fixation.

The foregut of the herbivorous Tammar wallaby, *Macropus eugenii*, produces significantly less methane than ruminants per unit of energy intake, and harbors a unique gut microbiota with, then uncultured, OTUs of the *Succinovibrionaceae* being the dominant members of the *Proteobacteria*. Pope et al. [92] used binned metagenomic assembly data, which yielded approximately 2 Mbp (of the near 3 Mbp genome size) to partially reconstruct the nitrogen and carbon utilization pathways, and antibiotic resistance, of the dominant *Succinovibrionaceae* group, WG-1. WG-1 was predicted to use starch as a carbohydrate source and the assembly included a urease gene cluster encoding all 13 genes required for urea transport and catabolism. Using this knowledge, they developed a defined medium, which contained starch and urea as the sole carbohydrate and nitrogen sources, respectively. The antibiotic bacitracin was also supplied in the medium because a putative bacitracin resistance gene was identified in the WG-1 assembly. Adopting this strategy, WG-1 was successfully enriched for from the wallaby digesta samples, and these were used to generate axenic cultures through dilution and plating on the enrichment medium. This work afforded a detailed characterization of WG-1 substrate utilization and fermentation in culture to further understand its contributions to lower methane emissions.

Metagenome sequencing permits the identification of specific substrates of putative novel species, then employed these to isolate new species through a cultivation method. Lugli et al. [93] utilized this method to isolate novel bifidobacteria from animal fecal samples. First, metagenomic data were assembled, then predicted genes compared to a glycosyl hydrolase (GH) database to assess the glycobiome of the bifidobacterial species. The authors predicted four glycans, consisting of arabinogalactan, pullulan, starch, and xylan, were carbon sources for these putative novel bifidobacterial species. Thus, cultivation experiments were performed using various chemically defined medium, containing a specific glycan, as indicated above, as its sole carbon source. As a result, 13 phenotypically different bifidobacterial isolates were cultivated which were able to metabolize the selected glycan, and two strains were novel bifidobacterial species [93]. Karnachuk et al. [94] cultivated a thermophilic spirochete from deep aquifers, represented by a strain, BY33, from a novel family of the order *Brevinematales*. Firstly, a novel MAG was identified from metagenomes of the deep subsurface aquifers. This genomic information suggested the presence of genes that encode enzymes that enable the utilization of starch, and maltose/maltodextrin ABC transport system for uptake of extracellular starch hydrolyzed products. Finally, enrichment and cultivation experiments were performed using modified spirochete medium with maltose and starch, and BY33 was isolated successfully. In the study by Renesto et al. [95], the genome of the pathogen, *Tropheryma whipplei* revealed metabolic deficiencies in amino acid biosynthesis. Nine amino acid synthetic pathways (for histidine, tryptophan, leucine, arginine, proline, lysine, methionine, cysteine, and asparagine) were absent in the genome, which suggested that *T. whipplei* acquires amino acids or their precursors from the external environment. Using this information, a comprehensive cell culture medium was developed that provided the missing amino acids and resulted in the isolation of three *T. whipplei* strains, previously cultivated from human cells and one new strain from a clinical sample. While this example was derived from genome sequence data, this approach can be similarly applied to SAG and MAG data derived from uncultivated samples.

An understanding of the optimal conditions for growth is also an important factor for the isolation and cultivation of bacteria. David et al. [96] reported a valuable tool that can predict the optimal growth temperature based on genomic information. The authors suggest that the same principle could be readily applied to other factors such as temperature, pH, salinity, osmolarity, or oxygen concentration. Knowledge of such factors may crucial for isolating microorganisms such as psychrophiles, thermophilic bacteria, salt-tolerant bacteria, and halotolerant bacteria from extreme environments.

#### Antibiotic resistance gene application

The antibiotic resistance phenotypes in microbiota can be directly linked to specific taxa and provide useful phenotypic information [97] that cannot readily be derived from culture independent studies. Metagenomics and network analysis were able to profile antibiotic resistance genes (ARGs) and their co-occurrence patterns in the microbiome [98–100] (Fig. 1). In this way, a variety of taxonomic groups were able to be detected and assessed for the phylogenetic distribution of antibiotic tolerance phenotypes. Mapping the antibiotic tolerance profiles among microbes allowed the targeted recovery of specific taxa with previously uncultured isolates. Rettedel et al. [101] used this approach to determine the phylogenetic distribution of antibiotic tolerance phenotypes for 16 antibiotics in the human gut microbiota. Using combinations of these antibiotics, they identified four isolates and two of them are novel species, belongs to the genus *Oscillibacter*. In the example by Pope et al. [92] above, bacitracin was used to help select for WG-1 because a putative bacitracin resistance gene was identified in the WG-1 assembly. Thus, antibiotic tolerance phenotyping provides useful in cultivation applications.

#### Stable-isotope probing guided RACS

Another isolation approach of new microbial cells is stable-isotope probing guided Raman-activated microbial cell sorting (RACS) [102], which yields live cells suitable for cultivation (Fig. 1). Metagenomic sequence data provides valuable information on traits such as primary metabolism, substrate utilization, oxygen requirements, resistance to antibiotics, which provide opportunities to define culture media and growth conditions for microbes [89]. Raman microscopy with deuterium isotope probing (DIP) has been demonstrated to identify the targeted bacteria with metabolic activity in specific medium [102, 103]. Thus, targeted bacteria can be isolated through the defined medium designed via MAGs or SAGs, including but not limited to specific substrate or antibiotics, based on labelling with the stable isotope deuterium during incubation with heavy water (D_2_O). D_2_O is an effective DIP probe for Raman detection of substrate shifted from the C-H band at 2000-2300 cm^−1^. When samples incubated in the designed medium, which contained the specific metabolic substrate and D_2_O, metabolic activated bacteria will generate C–D band in the single-cell Raman spectra (SCRS). Then, RACS helps for the isolation of these targeted bacteria according to SCRS [104].

Yi et al. [103] used RACS to detect metabolic activated antibiotic-resistant bacteria *in situ* from human gut microbiota. Fecal samples were cultivated in PBS supplemented with 40% D_2_O (v/v) and antibiotics (amoxicillin, cephalexin, florfenicol, tetracycline and vancomycin) for 24 h at 37 °C. They successfully sorted six samples including two amoxicillin-resistant bacteria (AmoxR), two cephalexin-resistant (CephR), and two cephalosporins-sensitive (CephS) bacteria. Kang et al. [102] applied this approach to sort bacteria involved in mucin degradation from the mouse colon. Colon contents were suspended in 50% D_2_O-containing PBS and porcine stomach mucin and incubated anaerobically. RACS identified a diverse consortium of bacteria, including several members of the underexplored family Muribaculaceae.

The RACS platform can be readily customized to sort cells according to other parameters, such as storage compounds [105], pigments [106], and other compounds [107], if they are represented by sufficiently large peaks in the Raman spectra of the microbial cells. For example, Yun et al. [107] classified five different types of bacteria isolated from the oral cavity based on ^13^C and ^15^N. These studies highlight the potential of Raman-activated cell sorting for identifying and isolation key players in targeted processes, in the age of omics.

#### Reverse genomics guided isolation

**Fig. 2:**
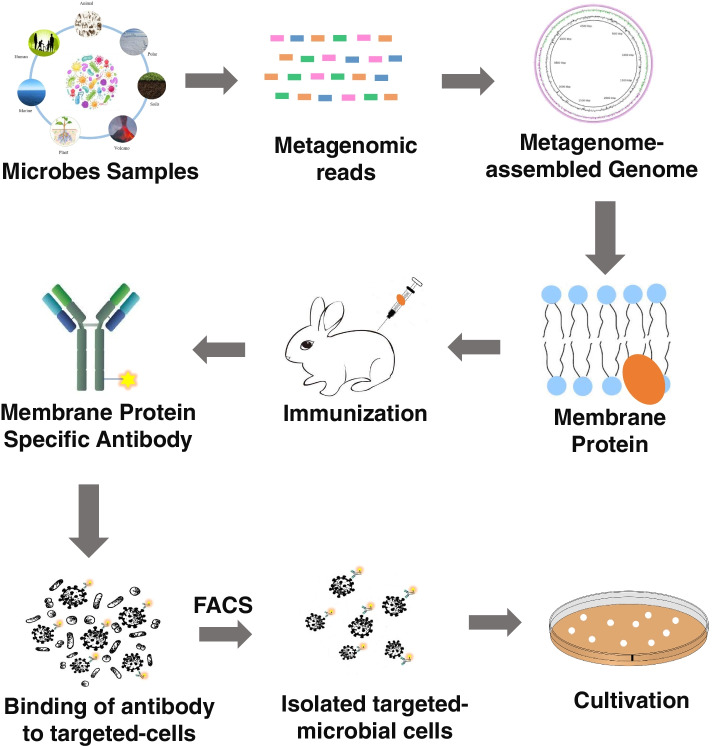
Flowchart demonstrating the approach of genome-informed antibody engineering reverse genomics for targeted isolation and cultivation of microbes

Cross et al. described a genome-informed antibody engineering approach to capture previously uncultured microbes of interest from complex communities, which they have described as reverse genomics [108]. The use of specific antibodies enables the efficient labelling of target cells while also maintaining their viability for cultivation (Fig. 2), unlike other labelling techniques. In brief, genomic data from the uncultured microbes of interest, which may derived from single-cell genomics as used by Cross et al., but could also be from assembled metagenomic data, are mined for genes predicted to encode membrane proteins with extracellular epitopes that are unique to the target microbe of interest. Antigen peptides are synthesized and used use raise antibodies, which are then fluorescently labelled. The antibodies were then allowed to bind to the complex microbiota samples, and the fluorescently labelled target cells were retrieved from the community using fluorescence activated cell sorting (FACS). The target cells maintained viability throughout this process and were used as inocula to successfully establish new cultures, though it is acknowledged that cultivation would only be achieved if robust cultivation conditions for the microbe of interest can be identified.

Cross et al. used this method to culture *Saccharibacteria* (TM7), a phylum belonging to the bacterial candidate phyla radiation [109], from human saliva samples, which represented three different species-level lineages of *Saccharibacteria*. SR1/Absconditabacteria, found in low abundance in human oral samples, were also brought into cultivation for the first time using the reverse genomics method [108]. These successes highlight the potential of reverse genomics for accelerating the cultivation of other uncultured microbes of interest, including archaea, especially those that are slow-growing, of low abundance and or rare species.

#### Gene-targeted isolation

**Fig. 3:**
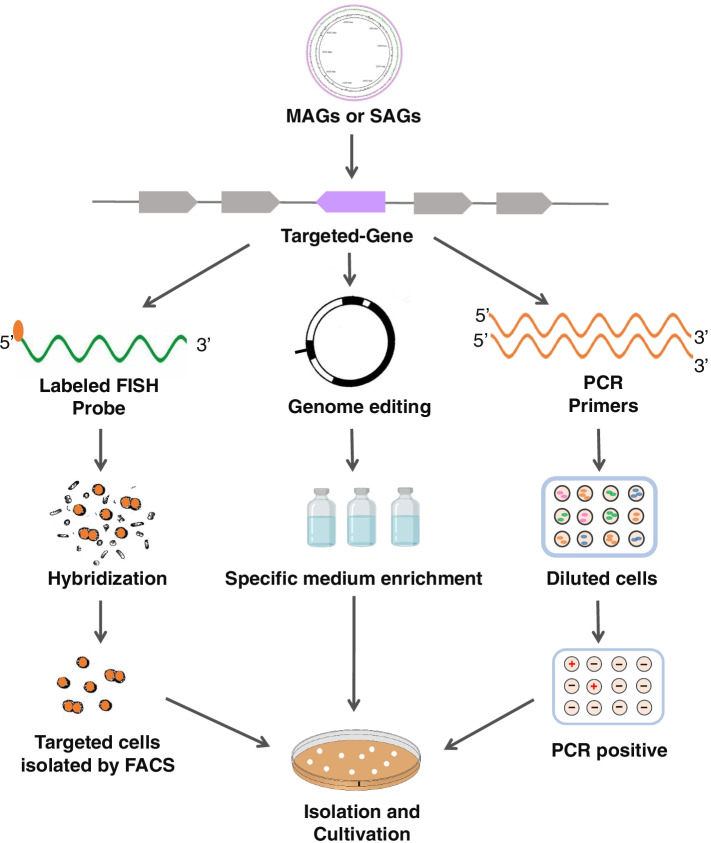
Overview the method of gene-targeted isolation of microbes

Metagenomic sequence data can be used to identify genes for the targeted isolation of microbes. Such genes may include functional genes of interest, for which specific variants may be revealed through metagenomic sequencing. Shotgun metagenomic sequence reads also have the potential to reveal rare 16S ribosomal RNA gene sequences, that may not be readily detected in amplicon sequencing datasets due to quality filtering steps that remove low abundance reads, or are derived from artefacts during the amplification step [110]. Ma et al. described a direct approach that cultivates, in a targeted fashion, microbes carrying genes of interest identified in metagenomic data. This method is carried out using a microfluidic device with nanoliter wells that allow microcolony growth derived from single cells, and is comprised of two main steps: identification of cultivation conditions for the target organism and isolation of the target [111]. The sample is diluted in growth medium so that at most, these is a single cell per well in the microfluidic chip, though most wells are empty in order to minimize the occurrence of more than one cell in a well. After cultivation, the chip allows each microcolony to be split into two, from one of which PCR for gene of interest is performed on to identify the cultivations conditions that allow growth of the target cells. Once cultivation conditions are identified, the cells are further applied to the chip and grown, then the microcolonies are PCR screened individually to identify the compartment containing the target, and then live cells can be retrieved from the corresponding well on the other half of the chip for scale-up cultivation (Fig. 3). Ma et al. [111] validated this approach by cultivating a bacterium from a human cecal biopsy, and cultured representative of a previously unidentified genus of the *Ruminococcaceae* family. This approach overcomes sampling bias from differential microbial growth kinetics, and the small size of the chip enables the use of growth stimulants available only in small quantities, and use in anaerobic environments to isolate strict anaerobes, such as those from gut environments. The difficulty of small volume cultivation methods is the supply of gaseous substrates. However, the new methods of enhancing and providing a homogeneous oxygen supply for droplet microfluidics could be applied to microplates [112].

One method that shows promise to capture target cells of interest using probe sequences derived from metagenomic sequence data is fluorescent in situ hybridization (FISH)-labelled samples that can be sorted with fluorescence-activated cell sorting (FACS) to enrich cells belonging to selected taxonomic groups [113]. Metagenomic sequence data can be used to design FISH probes to sort target microbiota carrying the genes of interest. However, standard FISH procedures require fixation of cell samples, rendering cells non-viable; hence, the cultivation of labelled cells is not possible. There have been recent advances to modify FISH procedures by removal of the fixation steps (live-FISH), which results in the ability to sort specific taxonomic groups of bacteria and subsequently culture them [113]. Tan et al. [114] demonstrated the use of FISH probes designed directly from short sequence reads obtained from metagenome shotgun sequencing dataset (R-probes), which were used on wastewater treatment plant samples. The R-probes were designed from ribotags, where the V6 hypervariable region tag sequence, which has a default length of 33 bp, served as template for FISH probe design and enabled successful enrichment of a novel bacterial taxon (UPWRP_1)assigned to the order Sphingobacteriales using Ribo_Unk1029_17 by FISH–FACS procedures. In some instances, probe design on the hypervariable region allowed differentiation to the species level. This approach, in combination with a live-FISH, could enable the cultivation of target species.

Some antibiotics function by directly binding to a specific bacterial surface structure, thus providing the opportunity to use these drugs as probes for bacteria [115]. Fluorophore-derivatized antibiotics probes retaining bacterial-binding ability have been a handy tool in isolating bacteria and have been utilized in tracking different bacterial species both in vitro and in vivo [116].

In addition, genome editing also can be used in isolation of new targeted microbes. The editing of specific genes based on MAGs sequence information can facilitate the design of growth medium such as that containing selective antibiotics or substrates, which enables positive selection of edited microbial cells with negative selection of unedited cells in the community (Fig. 3).

Rubin et al. [117] characterized and validated a general strategy for editing the genomes of specific organisms in microbial communities. They designed loss-of-function mutations of *pyrF* genes in *Klebsiella michiganensis* and *Pseudomonas simiae* by VcDART transposon. The mutations contained transposons carried two antibiotic resistance markers conferring resistance to streptomycin, spectinomycin, and carbenicillin. The organisms were successfully enriched to an abundance of > 99%. In addition, gene editing of lactose assimilation genes of target bacteria and provision of lactose as a sole carbon source in growth medium successfully enriched the targeted bacteria to the abundance of ~ 95%. The methodology of gene editing guided enrichment also had a high resolution that could distinguish different strains in the same species, such as *E. coli* subsp. 2 and 3 [117].

### Challenges and limitations of metagenome-guided microbial isolation

While there have been numerous studies that have successfully used metagenomic sequence data to guide the isolation and cultivation of microbes, there are still some challenges and limitations that are yet to be overcome to enable more widespread success.

Genome-informed antibody engineering provides an exciting new opportunity for the targeted cultivation of microbes, several limitations are recognized. Firstly, it may be not possible to identify suitable surface exposed epitopes as structural data on homologs from related species may improve the prediction of such regions, but are not always available. Next, expression levels of the target membrane protein in situ might also be too low for antibody capture. However, gene expression or proteomics data, if available, could be used to identify membrane proteins that are highly expressed. Finally, post-translational modification of the target epitope, such as glycosylation, may sterically hinder antibody recognition of their epitopes [118]. To overcome these challenges, several epitopes could be selected as antigens, or entire protein domains could be used, but this may come at the cost of specificity.

The targeted isolation of anaerobic microbes [119] may pose as additional technical challenges for some of the targeted isolation methods, such as those involving cell sorters. Due to their size, the use of cell sorters, in standard anaerobic chambers, is generally limited. The development of fluorescence-based cell sorters that can be used under anaerobic conditions would be valuable in this regard.

Once target organisms have been isolated, to maintain their growth continuously, a suitable growth medium and physicochemical conditions must be found. However, genome sequences alone often provide insufficient information for accurately determining all necessary requirements to grow a particular microorganism successfully, since the chemical composition of natural environments is often unknown. For example, Lavy et al. [120] designed a medium according to genomic data available for Candidatus *Poribacteria* sp*.* WGA-4E. Although metabolic properties of this phylum, such as possible utilization of urea as nitrogen source and assimilatory sulfate reduction metabolism, were deduced from genomic data [62], this specific medium under the conditions used did not result in the capture of *Poribacteria* in culture. Potentially the concentrations of medium components were suboptimal for *Poribacteria*, or the addition of signal molecules may have been required [120]. Complementing metagenomic approaches with other empirical methods, such as culturomics [19, 83], in situ culture [84, 85], single-cell isolation [86, 87], by factorial trial and error may be will help researchers to achieve cultivation.

The recovery of genomic data from target organisms of interest to gain insight into cultivation strategies, largely relies on their representation and effectiveness of DNA extraction methods, which common problems are incomplete cell extraction, cell lysis, or DNA degradation [121]. These may impact the representation of certain species in metagenome data. Single-cell genomics is an alternative option to reconstruct draft genomes of the target microbes [122, 123], but this requires specialist technical expertise.

## Conclusions

The rapid development of DNA sequencing technologies has unveiled the enormous variety of as-yet-uncultured microbes in nature. Now, the challenge is to use these data to support cultivation and explore ecological questions about the roles of microbes and microbiomes in their natural habitats. Metagenome sequencing has greater potential than providing new insights into microbiome function, as it also brings new opportunities for microbial isolation and cultivation. In particular, the long-read DNA sequencing technologies, greater depths of sequencing and advanced bioinformatic methods can improve the quality of genomic data from individual organisms and species via MAGs, enabling more accurate metabolic interpretation. These data support new culture medium development, genome-informed antibody engineering and gene-targeted cultivation, and a variety of microbes have been successfully isolated from different environments. The possibilities for microbial isolation guided by metagenomic and culture-independent sequence data are plentiful and can significantly increase the chances of successful cultivation for target organisms of interest. However, it is recognized that the processes for targeted cultiation are complex and currently require good comprehension of genomic data to predict cultivation requirements, or advanced technologies that are not readily available to all microbiology laboratories. However, such directed methods can decrease the overall time to isolate specific target microbes and bring them into culture. The challenge is to now use the abundance of culture-independent genetic data for high throughput targeted cultivation, which, combined with advances in cultivation methodologies, may lead to new breakthroughs in the capturing of the uncultured majority.

## Data Availability

Data sharing not applicable to this article as no datasets were generated or analyzed during the current study.

## Abbreviations

REFS: References
OTU: Operational taxonomic unit
MAGs: Metagenome-assembled genomes
NGS: Next generation sequencing
stLFR: Single-tube long fragment read
SAGs: Single-cell amplified genomes
HMMs: Hidden Markov models
GO: Gene Ontology
GH: Glycosyl hydrolase
ARGs: Antibiotic resistance genes
FACS: Fluorescence activated cell sorting
PCR: Polymerase chain reaction
FISH: Fluorescent in situ hybridization
RACS: Raman-activated microbial cell sorting
BGCs: Biosynthetic gene clusters
DIP: Deuterium isotope probing
SCRS: Single-cell Raman spectra
